# Care-experienced young people’s views and experiences of accessing general practice and dental services and attending health reviews in England: a qualitative study

**DOI:** 10.1186/s12875-024-02569-0

**Published:** 2024-08-29

**Authors:** Lauren Herlitz, Emily Ashford, James Baldwin, Claire Powell, Jenny Woodman

**Affiliations:** 1 NIHR Children and Families Policy Research Unit, Population, Policy and Practice, UCL Great Ormond Street Institute of Child Health, 30 Guilford Street, London, WC1N 1EH UK; 2 Thomas Coram Research Unit, UCL Social Research Institute, 55 Gordon Square, London, UK; 3 Toucan Theatre Limited, 7 Bell Yard, London, WC2A 2JR UK

**Keywords:** Looked after, Out-of-home care, Primary care, General practice, Dental practice

## Abstract

**Background:**

Children in care and care leavers have worse health outcomes than their peers without care experience. This study addresses an evidence gap in exploring care-experienced young people’s views and experiences of accessing general practice and dental services and attending health reviews in England.

**Methods:**

We conducted a qualitative study using podcasting as a creative medium. We recruited young people from two sites: one in South England (A) and one in greater London (B). We held two paired discussions in site A and two focus groups in site B, with 14 participants in total. Participants were aged between 13 and 22 years and were diverse in gender, ethnicity, and care experiences. Data were analysed thematically using candidacy theory as a theoretical framework.

**Results:**

Mental health was a prevailing concern for participants, but general practice was not considered a place to discuss it. Most participants reported distant relationships with primary healthcare professionals and considered opening-up to a professional to be risky, for example, it could result in an unknown/unwanted outcome. A lack of time and personal connection in appointments, and experiences of feeling judged, dismissed, or misunderstood, hindered young people’s ability to disclose mental health or relationship concerns. Participants reported variation in the timeliness and location of services, with salient examples of extensive waiting periods for braces. Participants perceived annual health reviews to be largely inconsequential.

**Conclusions:**

Any primary care presentation by a care-experienced young person should trigger additional professional curiosity. To build rapport and trust, professionals should not underestimate the power of active listening, being reliable and honest, and small acts of thoughtfulness, for example, ensuring medical letters are provided promptly. Carers and other trusted professionals should help care-experienced young people to understand the role of primary care and support them with access. Health reviews may not be of value to all young people in care. Further research is needed to examine primary healthcare access for care-experienced young people with significant safeguarding and healthcare needs.

**Supplementary Information:**

The online version contains supplementary material available at 10.1186/s12875-024-02569-0.

## Background

In England, ‘looked after’ children are those that have been removed from their birth parents’ care and placed in the care of the local authority (also known as ‘children looked after’, ‘in care’, or ‘out-out-home care’) [1]. Around 26 in every 10,000 children aged under 18 in England enters care each year [2]. In 2022/23, over 83,000 children were looked after and numbers of children in care have been rising since 2008 [2]. Looked after children who have been in care for a period of 13 weeks or more spanning their 16th birthday are known as ‘care leavers’ and are also entitled to state support up to the age of 25 [3].

Care-experienced children and young people have been identified by NHS England as a group experiencing health inequalities [4]. Evidence from cohort, cross-sectional and qualitative studies suggests that care-experienced children and young people have poorer mental health, higher substance use, and more relationship difficulties than their peers who have not been in care [5–9]. A systematic review found that care-experienced children and young people experienced poor oral health, and had limited oral health self-care and knowledge [10]. Cross-sectional studies in Sweden and the US have found children entering care had unmet health needs [11] and children placed in care due to maltreatment were more likely to have complex health needs [12]. Poorer health outcomes have been attributed to: adverse childhood experiences prior to entering care affecting children’s health and their ability to self-care, and impacting on their willingness to engage with healthcare services; poor health behaviours before entering care (e.g. high sugar diets and low dental hygiene); poor experiences of care placements; placement instability affecting recognition of health needs; and lack of support received on leaving care [13–18].

Access to healthcare services may also affect care-experienced children and young people’s health outcomes. In England, statutory guidance requires local authorities to arrange a health assessment for children entering care and to organise regular health reviews for looked after children [19, 20]. Health assessments should address a child’s state of physical, emotional and mental health, health history and its impact on development, and explain existing arrangements for medical and dental care [21]. Recent national figures indicate all children and young people in care are receiving initial health assessments and most (89%) are up-to-date with annual health reviews; immunisation rates are slightly lower and dental check-ups are higher than children in the general population [2, 22, 23]. However, figures at local authority level reveal significant variation in rates between areas [2] and a parliamentary inquiry found access to mental health services to be inadequate, with health assessments failing to pick up on children in need of specialist care and support [24]. Studies from the US and Australia suggest access can be compromised by carers and residential staff not prioritising healthcare or lacking health literacy, insufficient/delayed funding, delays to receiving a health insurance number, difficulties gaining consent for medical procedures, poor care co-ordination, and long waiting lists [17, 25–27]. A systematic review found that care-experienced children and young people were less likely to access dental care for reasons including carers/professionals not knowing how to secure dental care, difficulties obtaining dental health histories, placement changes, and children’s anxiety over treatment [28].

Few studies have been conducted on care-experienced young people’s views and experiences of primary healthcare services [29]. Qualitative studies in the UK and Australia found that care-experienced young people had a poor understanding of the healthcare system, including differences between children’s and adults’ services [25, 30, 31]. In one study, young people reported feeling judged, not being asked enough questions, or not being listened to by medical professionals [25]. In another, young people highlighted difficulties accessing mental health support because of feelings of embarrassment, guilt, or fear, and stigma around mental health [32]. Qualitative studies of care leavers’ views have found that young people appreciated healthcare workers building a caring and consistent relationship with them, speaking to them about the transition to adult services, and supporting them with other needs (e.g., writing to housing services) [31, 33].

In summary, care-experienced children and young people have poorer health outcomes than their peers, and access to healthcare services may be affected by the circumstances related to their care status. However, there is limited evidence regarding young people’s perspectives of access to healthcare services, particularly prior to leaving care. This study aimed to identify whether there are opportunities to improve the experience of primary healthcare services for care-experienced young people with a view to improving health outcomes. We focused on access to primary care as it is the front door of healthcare services and has a key role in reducing health inequalities [34]. We asked, “What are care-experienced young people’s views and experiences of accessing general practice and dental services, and attending health reviews?”

## Methods

We conducted a qualitative study using the arts-based medium of podcasting to explore participants’ views and experiences. We defined access as the opportunity to identify healthcare needs, to seek, reach and use healthcare services, and to have healthcare needs met [35, 36]. Additional file 1 provides further methodological details, including a reflexivity statement.

The study received approval from the University College London Research Ethics Committee (17893/004).

### Sampling and recruitment

We worked with facilitators of established care-experienced groups based in two council areas: one in South England (site A) and one in Greater London (site B). In 2022, site A had a much higher rate of looked after children than regional and national rates, and more than double the rate of site B. Sites were selected opportunistically through existing professional contacts.

The local facilitators had longstanding relationships with the young people in their groups and were responsible for: selecting potential participants; sending out to young people and their carers links to online information sheets, consent forms and a ‘consent explainer’ – a plain English breakdown of the consent form; and supporting the session facilitation. To assist with recruitment in site A, a care-experienced youth participation trainee created a short study information video with us. In site A, the council required that a participants’ social worker (or their manager or team leader) also provided consent.

Young people were eligible to take part if they were: aged between 13 and 25 years; were members of one of the youth groups; available to participate on one of the dates proposed; and local facilitators considered them to be emotionally able to engage and share their experiences safely in the group setting. We aimed to recruit three groups of four to six participants for each focus group (up to 18 individuals in total); two groups with ages 13–17 years, and one group aged 18–25 years. The age groupings were determined by the existing age ranges of the council’s established groups (typically a group for children in care and a separate group for care leavers).

Informed consent to participate was obtained from all study participants, their carers if participants were under 18, and from a social care representative if required (as mentioned above). One young person who had consented in site B decided they did not want to participate on the day.

## Data collection

LH and JB conducted the workshops using podcasting to engage participants in sharing their experiences of healthcare access [37, 38]. Podcasting is the process of creating a digital audio story or conversation that can be listened to on-demand. We selected it as the creative medium as podcasting can be used to enhance participants’ collaboration and reflection, and to give a platform to young people that are seldom heard [37, 39, 40]. For example, podcasting has been used to hear the first-hand accounts of migrants and young sibling carers [41, 42]. As an output, podcasts can create a space for an audience to engage emotionally with a subject and understand the complexity and meaning of people’s experiences [37, 43]; it can challenge dominant representations of underserved groups and create interest and action for change [42, 44]. Additional file 1 contains information on how we produced the podcast output and where it can be accessed.

Workshops in sites A and B took place in November 2022 and April 2023 respectively and were audio-recorded. The workshop consisted of four core parts: an introduction, familiarisation with the microphone and equipment, a true/false game and discussion, and participants’ ideas about healthcare improvements. The option to script a monologue about a young person was used in one workshop where we thought that participants would engage with the activity. Workshops took place in youth spaces in council buildings and lasted 2–2.5 h with lunch or dinner provided (see additional file 1). Participants were given a £50 voucher as thanks for participation.

### Data analysis

We thematically analysed the transcripts from the workshops [45]. After familiarisation with the data, EA and LH conducted line-by-line coding using NVivo 12 software. Each code’s data were checked for consistency of interpretation and emerging themes were discussed by EA and LH. LH then applied candidacy theory as a theoretical framework to enhance the explanatory power of the analysis [46, 47]. The theory “describes the way in which people’s eligibility for medical attention and intervention is jointly negotiated between individuals and health services” [48]. It proposes that accomplishing access requires individuals to perceive themselves as an appropriate candidate for a service and carry out work to negotiate access; and the amount, difficulty, and complexity of that work can be a barrier to receiving care [48]. Candidacy theory has been used to explain healthcare access for underserved populations [49, 50], with some applications in youth populations [51, 52].

**Fig. 1:**
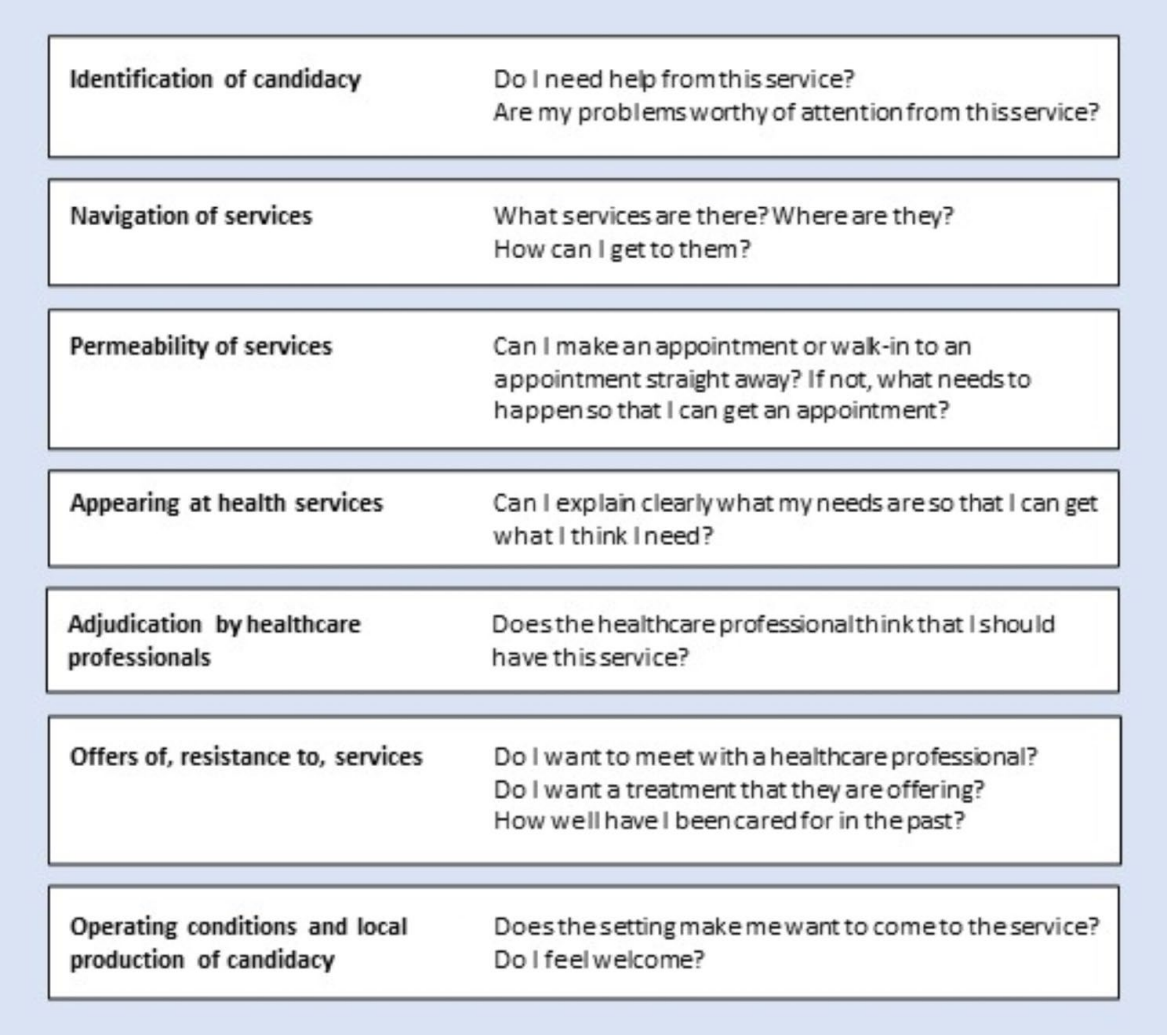
Adapted description of Candidacy Theory, adapted from Dixon-Woods et al. (2006) [48]

LH translated the theory’s seven features of candidacy into plain English to match the kind of language used by study participants (see Fig. 1). LH applied the theory deductively to the emerging themes, examining whether themes were describing features of candidacy. LH re-examined the data to consider whether two features that had not been identified in emerging themes had been overlooked or included under a different feature (see additional file 1). In the final analysis, we found the data supported six of the seven candidacy features (with ‘permeability’ absent). LH discussed theme development with the wider team, and after iterations, the team agreed the final themes.

## Results

### Sample characteristics

Two paired discussions and two focus groups (hereafter referred to as ‘workshops’) were held in sites A and B respectively, with 14 participants in total (see Table 1). Participants were diverse in gender, age, ethnicity, and care experiences (Table 1; age has been presented in ranges to preserve anonymity). We did not ask about directly about participants’ care history beyond the demographic information we collected, but from our discussions it was clear that some participants had been in one stable placement throughout their childhood, while others had had multiple placements; some had entered care in their teens, with one young person coming to the UK as an unaccompanied minor. Most participants under 18 were living with foster carers and some lived with extended family. A few young people reported having contact with their biological parents. Two sets of participants in workshop C and in workshop D were siblings.

**Table 1:** Participants’ characteristics

ID	Gender	Age	Ethnicity	Age first looked after	Single or multiple placements	Workshop
1	Female	13–14	White	12	Single	A
2	Female	15–17	White	5	Single	A
3	Male	18–22	Mixed	6	Multiple	B
4	Male	15–17	White	3	Single	B
5	Non-binary	15–17	Mixed	14	Single	C
6	Female	18–22	Black	16	Single	C and D*
7	Male	13–14	White	5	Single	C
8	Female	15–17	Mixed	11	Multiple	C
9	Male	13–14	Mixed	10	Multiple	C
10	Female	13–14	Black	9	Single	C
11	Female	13–14	Black	8	Single	C
12	Female	18–22	Mixed	5	Single	D
13	Male	18–22	Mixed	4	Multiple	D
14	Male	18–22	White	2	Multiple	D

*Participant_6 is a regular attender of both groups and participated in both workshops

### Views and experiences of primary care

**Fig. 2:**
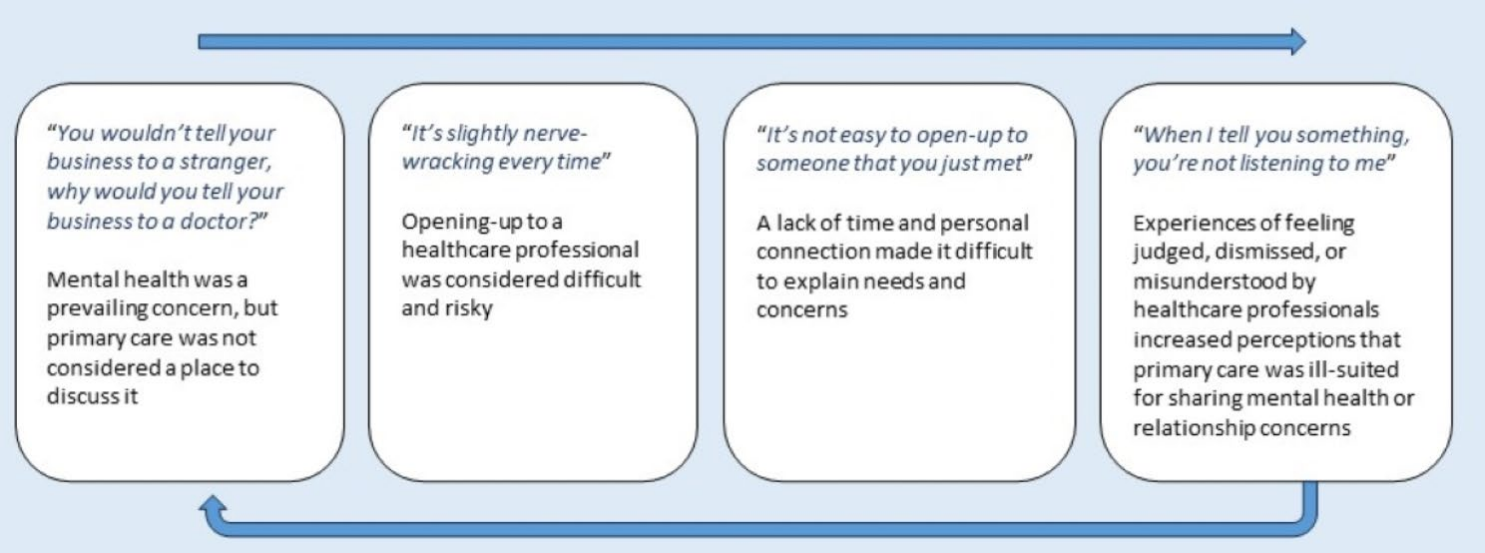
Participants’ reticence to seek healthcare support for mental health or relationship concerns

**Fig. 3:**
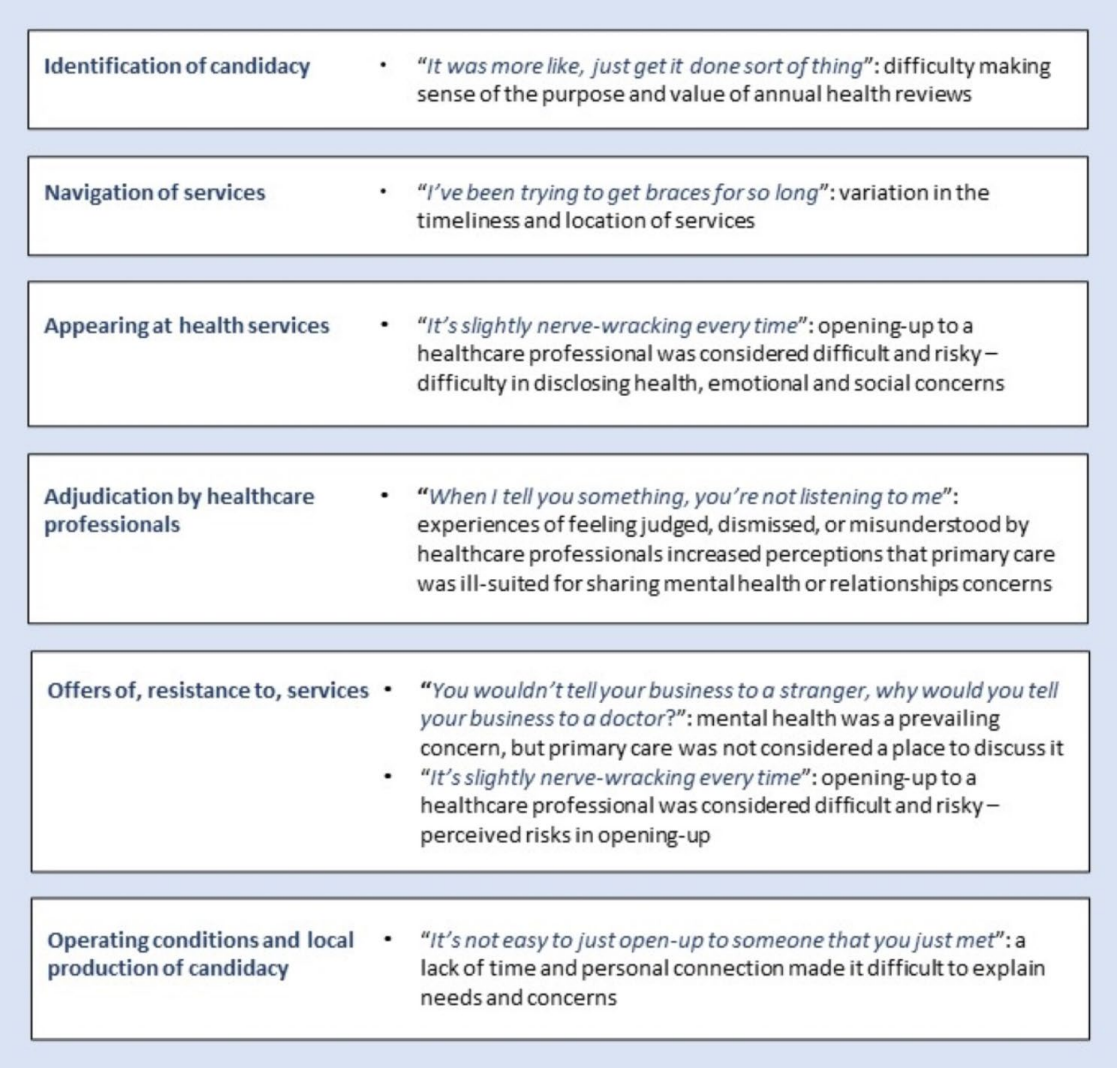
A summary of each theme’s connection to candidacy theory

We constructed six themes on care-experienced young people’s views and experiences of primary care. The first four themes explained participants’ reticence to seek healthcare support for mental health or relationship concerns and were connected in a feedback loop (see Fig. 2). Figure 3 summarises how the themes relate to features of candidacy theory.

#### *“You wouldn’t tell your business to a stranger*,* why would you tell your business to a doctor*?” : mental health was a prevailing concern, but primary care was not considered a place to discuss it

Although mental health was highlighted by all participants as a salient health concern for them, and they spoke cogently about the impacts of poor mental health, most participants thought that a GP was not a suitable person to speak to about mental health or relationship concerns. This theme aligned with the candidacy feature of ‘offers of, resistance to, services,’ describing participants’ reticence to receive emotional support from primary healthcare professionals.

Some participants thought that talking to young people about mental health or relationships was not part of a GP’s role or that they would be disinterested; some participants explained that a GP’s role was to diagnose and treat problems rather than to listen and understand their struggles. Several participants thought GPs lacked knowledge of how to support young people that had experienced trauma. One participant, who was an unaccompanied minor, said she thought a GP would have nothing to offer that could help her with the sadness she felt from being separated from her mother:

*I’m living without my family*,* my parents and then I don’t think sometimes I can live even without my mum*,* if I go to my doctor*,* if I told him that I have a problem in my knee… I believe that they can treat it but… they cannot make my mum to come here and then meet her. * Participant_6

When participants discussed who they would prefer to speak to about their mental health, many commented that they would rather see a specialist/therapist who had expertise in mental health and with whom they could form a relationship through regular contact. Many young people also reported that they would prefer to talk to family and close friends about their worries because they trusted them and knew them well, and a couple of participants said they would open-up to another trusted adult (e.g., their carer or a social worker).

#### *“It’s slightly nerve-wracking every time”*: opening-up to a healthcare professional was considered difficult and risky

Many participants described the difficulty and potential risks of opening-up to a healthcare professional. We constructed two sub-themes: (1) difficulty in disclosing health, emotional and social concerns, aligning with the candidacy feature of ‘appearing at services,’ young people’s ability to explain their needs in a way that enabled them to access the right care; and (2) the perceived risks in opening-up, supporting the candidacy feature of ‘offers of, resistance to, services’.

### Difficulty in disclosing health, emotional and social concerns

**Fig. 4:**
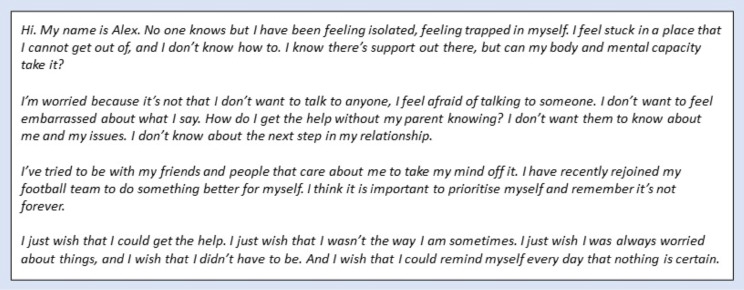
Participant-created monologue presenting a young male’s difficulty in opening-up

Many participants described the ‘*struggle’* they had in opening-up to other people about their health, emotional and social concerns, matters that they considered to be very private or personal. Several young people said poor mental health could leave you feeling ‘*trapped’* inside yourself. Figure 4 contains a short monologue created by two participants describing these feelings.

Several young people said that it was difficult to talk about their emotional or social needs with GPs, for example, the emotional strain of wanting to have more contact with a birth parent. One participant explained the feeling of having to look after themselves after being separated from their birth parents, making it difficult to know who to confide in:

*It’s like you have to take responsibilities for yourself now because you’re not living with your parents*,* it’s like you can’t say anything you would tell [to] your parents*. Participant_8

Two participants described how they were unable to express their fear of pain and the uncertainty of dental procedures when they were at the dentist because they felt it was expected by the professional that they would just endure the procedures without a say in how they were carried out.

Participants had different views on whether being accompanied by their carer in an appointment was a help or hindrance to disclosure. Participants were unsure of when a young person could start seeing a doctor by themselves; some believed there was a set age (16 or 18 years) while others thought it depended on maturity (being able to calmly communicate your concerns) or a combination of both. Two participants (aged under 16) reported that they would feel safer in GP appointments with their carer, one of whom also thought that a GP’s enquiries would be more thorough if their carer was present. Two participants (aged over 16) preferred to see a healthcare professional alone because they felt uncomfortable to speak openly in front of their carer. One participant (aged over 16) reported that they would prefer to attend their health review unaccompanied but were not aware if they could request this. One participant aged 18 + said that they had been more able to talk to their GP when they had reached 16 because there was a greater need to, with changes occurring both physically and in their transition to adulthood.

### Perceived risks in opening-up

Participants reported that disclosing concerns could make them appear vulnerable or expose them to judgement or criticism, for example, raising a trivial issue when other patients had genuinely serious problems. It could result in an unwanted/unknown outcome, such as the doctor speaking to their carer or another professional without informing them. Participants noted it could potentially make the problem worse, for example, by increasing stress for others, or lead to embarrassment. Two participants highlighted the counter argument that there was a risk in a problem worsening if nothing was done.

Most participants recognised that healthcare professionals would need to report safeguarding issues to children’s social care. When asked what would be considered a safeguarding concern there were varied responses: issues with family members, if a young person were in danger or were self-harming. Several participants commented that they were unclear on what constituted a safeguarding concern and so it was a risk to disclose concerns without being sure of the outcome:

*They say it’s confidential until like it gets really bad*,* like sometimes you don’t know what counts as really bad… sometimes you could say something and they might tell your carer*,* even though you don’t think it’s that bad. So… I don’t know what I can and can’t tell them*,* like what the limits are*. Participant_5

#### “*It’s not like easy to just open-up to someone that you just met*”: a lack of time and personal connection made it difficult to explain needs and concerns

The nature of consultations at general practice and dental services constrained young people’s ability to explain their needs, supporting the candidacy feature of ‘operating conditions and local production of candidacy.’ Participants’ willingness to disclose concerns was affected by a lack of personal connection with a healthcare professional. Regularly seeing the same professional was a preference for most participants so that they could get to know one another and develop ‘*a mutual respectful relationship*,’ but it was not the reality; however, two participants described how they had known their GP for several years and felt able to have difficult conversations. Most participants described how it took time to build rapport with healthcare professionals and the prospect of opening-up to a new person could be daunting.

*Say if it’s the first session and they’re saying*,* ‘Oh this child’s not opening [up] already*,* I want her to open up*,*’ it’s just like you’re kind of pressuring a child to open up*,* they might not say it to the child’s face*,* they might just say it to the child’s parents*,* it might be like 4 or 5 sessions or 10 sessions for the child to open up because maybe that child has trauma of trusting people.* Participant_11

Some young people reported feeling frustrated at having to repeat their concerns multiple times to different professionals. Two participants reported that a change in foster placements meant that they had to attend different GP and dental practices which resulted in losing the existing connections that they had made.

Some participants also highlighted the importance of the gender and ethnicity of the healthcare professional in creating a connection. A few reported that they would prefer to see a professional of the same gender because it made them feel physically safer (i.e., a young woman preferring a female doctor) or emotionally safer to disclose their concerns. One participant highlighted that they would prefer a therapist of the same ethnicity because they thought the professional would better understand their experience of being marginalised:

*It’s like talking to people about what it is to be black is kind of hard. When I think about it really*,* if I had a therapist who is black as well or kind of understands what racism feels like*,* it’s going to be more easy for them to sympathise with me than it is for a white therapist because they have privilege*. Participant_11

Most participants recounted experiences of feeling rushed in their appointments with their GP, “*it’s like rush hour for them*” (Participant_9) and reported that care-experienced young people may need and benefit from being given extra time to disclose concerns. Some participants felt that they had not had a chance to explain their worries properly or that the GP was not fully engaged in their consultation and perhaps their mind was elsewhere:

*It’s just a quick in and out thing. Like they just see you for your issue*,* they’re just prescribing medication and then they just send you out. There’s not enough time to like actually have a proper convo with them*… Participant_13

Several young people recounted experiences of being hurried through their dental care, leaving one participant in pain and needing further treatment, one in pain during lengthy surgery and feeling unable to ask for a break, and another switching orthodontists because the procedure had not been properly explained to them and they were too anxious to complete the treatment.

Two participants described how care leavers might need extra support, warmth and time from reception staff and healthcare professionals when they started to attend healthcare settings independently, as they had to learn to navigate many new experiences at once.

#### “*When I tell you something*,* you’re not listening to me”: e*xperiences of feeling judged, dismissed, or misunderstood by healthcare professionals increased perceptions that primary care was ill-suited for sharing mental health or relationship concerns

To enable young people to disclose their health concerns, participants reported that they needed to feel ‘*safe’* or ‘*comfortable’* and know that a professional would listen to them with interest and care and would not judge them. Several participants expressed firm normative beliefs about the behaviour of healthcare professionals, including that they must: help with both physical and mental illness; ensure your safety; refrain from passing judgement; listen to their patients:

*About certain things doctors and nurses aren’t allowed to question or query or judge you for something because actually at the end of the day it’s a profession. Their job is to help you and support you through it in the best way they can*. Participant_4

However, normative beliefs largely did not match participants’ actual experiences; many reported that professionals had not taken their views seriously or made judgements about the treatment they required without fully understanding their needs, aligning with the candidacy feature of ‘adjudications’. Some participants had felt that they had been given medication as a temporary solution rather than the GP taking the time to investigate further. While two participants described a trusting relationship with their GP, many young people reported feeling disregarded or belittled. Two participants (aged under 16) described how a GP had dismissed their difficulties as typical transient adolescent problems instead of comprehending that they were related to trauma experiences:

*They will all say like you’re going through a phase where like it’s like a normal teenage phase… but if you actually go back into the past*,* for example*,* of what I’ve been through as well as a young child*,* then this is not just a phase*,* this is just a like… It’s like a scar.* Participant_8

*…kind of hard talking to your doctor about it because they’re just going to say*,* ‘Oh no*,* you’re just going to feel like this*,* it’s like… just temporary*,*’ ‘Oh you’ll just get over it*,*’ and stuff and it’s like sometimes it feels like they’re not even listening to you.* Participant_11

Experiences and perspectives on judgement were discussed in each workshop. Many participants described feeling alert or vigilant to potential judgement from GPs about their behaviour or their presenting health issue, and carefully read body language and verbal cues. One participant insightfully explained that their life experiences had made them more sensitive to negative interactions:

*You do put your guard up massively and for people that are in care a lot it’s even worse if you get let down once*,* it’s like the whole world’s been thrown at you but really someone probably just couldn’t help you out that day.* Participant_3

#### “*I’ve been trying to get braces for so long*”: variation in the timeliness and location of services

When participants had decided to seek help, they had mixed experiences relating to the timeliness and location of services, chiming with the candidacy feature of the ‘navigation of services’. Many participants described difficulty getting a GP or dental appointment or being offered appointments that were several weeks away. Several participants described the frustration they experienced after having to wait years for dental care or to have their braces put in.

*I was waiting to… get braces in like Year 7*,* but I didn’t get them till like Year 10…. They just kept saying that the list for people that wanted braces was… full… I wanted them because I needed them*,* and I used to get bullied… because of my teeth*. Participant_12

In contrast, one participant thought access to medical services had been easier for them because they were prioritised as a looked after child. One participant who was a care leaver said they had never experienced difficulties with obtaining a dental appointment, however, they had not attended the dentist in several years due to cost.

Participants had different opinions of how easy it was for them to physically access primary care services, depending on whether their carer could take them, if it was in walking distance or whether they had access to, or the money for, public transport. A few participants indicated that ease of access affected how likely they were to attend, and could be a barrier to their needs being met:

*You’ve got the worry of going to the doctors in itself and then the worry of the travel itself*,* so you’ve then got a lot of pressures that are then building up to then the point at which you probably just think “you know what*,* I’d just rather not go.* Participant_3

#### “*It was more like*,* just get it done sort of thing*”: difficulty making sense of the purpose and value of annual health reviews

We explored participants’ experiences of annual health reviews in the workshops. This theme aligned with the candidacy feature of ‘identification of candidacy,’ as young people were reflecting on why they might need this service. Most participants reported that their reviews were not something that they were actively involved in, rather something that was mandatory and unproblematic but rarely useful. Aside from one young person who received health reviews in school, there was little sense that participants saw the same healthcare practitioner for their reviews. Rather than comprehensively reviewing their physical, emotional and mental health, there was a perception across participants that the health reviews largely focused on checks of height, weight, and heart rate, and in some cases, general questions about how things were going.

Most participants found it hard to make sense of the purpose or value of health checks; one participant thought the measures of height and weight was perhaps something a biological parent would have carried out, and two participants thought perhaps a young person would be put on a weight plan if they were overweight. A few participants appreciated the opportunity to have an annual health check “*because there might be something wrong*” (Participant_1) and because it was an opportunity to raise a concern. However, one participant found the checks annoying as they interrupted their free time at school and they considered themselves healthy, and another young person expressed frustration that if they raised any genuine health concerns, they were referred to their GP (perceiving the review as somewhat meaningless).

## Discussion

### Summary

Care-experienced young people reported their reticence to seek healthcare support from primary care, particularly for mental health. Trusted relationships were critical to their willingness to disclose concerns. Most participants did not have a long-standing relationship with their doctor – and were conscious of the risks of opening-up, including wasting professional time for minor concerns and information being shared with a carer or another professional. Experiences of feeling judged, being misunderstood, or dismissed discouraged participants from future help-seeking, resonating with findings from a review of children and young people’s access to primary care [53]. Participants explained that young people needed to feel listened to and given enough time to express their needs, findings reported in other studies of young people’s healthcare experiences [25, 54–56]. While young people in general may prefer continuity of care and clarity over confidentiality [54–57], it is even more important for care-experienced young people because experiences of trauma and broken trust, and transient relationships have strengthened resistance and wariness to sharing private concerns [28, 58]. Participants also reported other barriers to healthcare access, for example, long waiting times for dental care and difficulties expressing their needs in hurried appointments.

### Implications for research and practice

Several participants explained that trauma, separation from birth parents, and being in care were experiences that made them feel different from their peers without care experience. They were uncertain about who could help them and who they could trust to ask. This indicates that carers and other trusted professionals, e.g., youth workers and social workers, need to help care-experienced children and young people to understand the role of primary care, including that general practice is a gateway to accessing mental health, sexual health and other support services, and support young people to access healthcare when they become aware of unmet needs [30, 57, 59]. Many participants also expressed strong normative beliefs about the positive qualities of the healthcare profession, in spite of negative experiences, suggesting young people may be in a sensitive period of development where good healthcare experiences – characterised by empathy, transparency, reliability – can set a positive direction for future help-seeking [60–62].

Participants were mindful of the perceived risks in opening-up to professionals; speaking to a doctor or another practitioner was not a decision they made lightly. Consequently, professionals should assume that a care-experienced young people attending an appointment means that they have an issue that needs to be given space and time, even if they appear reticent to speak or engage. Care-experienced young people often have histories of poor healthcare prior to entering care and difficulty trusting adults, and may have witnessed poor health and difficult healthcare experiences within their own families (e.g. as a result of substance abuse or violence) [11, 13, 63]. This underpins the argument that continuity of care is clinically appropriate for care-experienced young people to enable them to disclose their concerns and have their healthcare needs met, as recommended by National Institute for Health and Care Excellence (NICE) guidelines on healthcare for children and young people [64]. The guidelines also recommend that looked after children and care leavers should be treated as a group with specific health needs, with healthcare professionals taking particular care in communicating and coordinating care [64]. While new primary care roles such as the children and young people’s mental health practitioner may improve the supply of mental health support, they may further diminish continuity of care for vulnerable young people for whom an established relationship is a prerequisite for information sharing [65]. Further research is needed on how to effectively achieve continuity of care for vulnerable children and young people and whether this leads to improved healthcare access and health outcomes.

Our study suggests that professionals, including reception staff, should take particular care to listen to each care-experienced young person who makes and attends an appointment, as there may be higher thresholds for help-seeking among this group as well as opportunities to set positive views of healthcare. Although medical professionals, including dental care practitioners, would benefit from training on trauma-informed care and children and young people’s mental health [28, 58, 66], the power of active listening, being reliable and honest, and “small acts of thoughtfulness” [67] (e.g. ensuring medical letters and records are provided promptly) should not be underestimated [60, 67]. Rotenberg’s [60] conceptual framework of the three bases of children’s interpersonal trust – reliability, emotional trust, and honesty – encompasses participants’ accounts of trust in healthcare professionals, and descriptions of trusted relationships given by other looked after children in a qualitative study of their perspectives on what matters to their emotional wellbeing [67].

Our study explored participants’ perspectives on their annual health reviews. Participants found it difficult to make sense of the value of the reviews, as they mostly recalled the review’s focus on height and weight. Further research is needed to explore young people’s, carers’, healthcare professionals’ and social workers’ perspectives on whether health reviews are appropriately tailored to young people’s development and level of health need to ensure they are genuine opportunities for adolescents’ needs to be met over and above usual services. While reviews might be valuable for some young people, offering this specialist service to all young people in care could potentially lead to unintended harms: it could reduce opportunities for young people to understand the universal healthcare system lowering health literacy when young people leave care, and it may also lead to their disengagement if they perceive themselves as healthy and want to be treated like their peers [25, 30, 68].

Candidacy theory strengthened our analysis by enabling us to draw out and articulate key themes, particularly related to operating conditions of healthcare provision and young people’s difficulties in communicating concerns, and to explain the negotiation of access between young people and services. It helped us to place our participants’ experiences within a wider body of literature on access to healthcare which shows that although patient experience is vital to improving access, particularly for vulnerable children and young people, policy interventions have predominantly focused on improving service supply [53, 69].

### Strengths and limitations

Our sample was diverse in gender, age, ethnicity and care experiences. Using podcasting as a creative medium during the data collection engaged participants and enabled us to create a research output that gives a voice to seldom heard young people which complements this written manuscript. While our sample was diverse, the participants were largely healthy (only two participants reported regular health check-ups) and were fluent in English. Our findings may not be representative of care-experienced young people with chronic and/or complex health needs, nor may it be representative of young people with particular safeguarding and healthcare needs, for example, unaccompanied minors and trafficked young people. None of our participants were in residential care, who may have different experiences of primary healthcare. Further primary research on healthcare experiences is needed with these groups of young people.

## Conclusion

The study findings suggest three key implications for working with care-experienced young people: (1) any presentation by a care-experienced young person should trigger additional professional curiosity and efforts to build rapport and trust, (2) carers and other trusted professionals need to help care-experienced young people understand the role of primary care and support them with access, (3) health reviews may not be helpful for all care-experienced young people, particularly if they are not fully informed of their purpose or told their right to refuse participation. Further research is needed to examine the healthcare experiences of care-experienced young people with significant safeguarding and healthcare needs.

### Electronic supplementary material

Below is the link to the electronic supplementary material.

Supplementary Material 1

## Data Availability

Data not available due to privacy/ethical restrictions.

## Abbreviation

NICE: National Institute for Health and Care Excellence
