# Supplementary material for: Care-experienced young people’s views and experiences of accessing general practice and dental services and attending health reviews in England: a qualitative study
Source: BMC Prim Care. 2024 Aug 29;25:318. doi: 10.1186/s12875-024-02569-0 (PMC11360303; doi:10.1186/s12875-024-02569-0)
Supplement: Supplementary file 1 — Supplementary Material 1 [file 12875_2024_2569_MOESM1_ESM.docx]

# Additional file 1: Further methodological details

**Reflexivity statement**

LH is a White British, female PhD and an experienced qualitative researcher, with previous experience of conducting interviews and focus groups with underserved young people. JB is a White British, male, and an experienced creative facilitator of podcasts and theatre, who had previously worked with care-experienced YP and other underserved YP and adults. LH and JB are in their forties. EA is a White British, female PhD student, in her twenties, with experience of analysing qualitative data. LH and JB collected the data and LH and EA conducted the analysis.

LH and JB expected that the YP might be guarded about talking about themselves and their health, and might be put off by the facilitators’ resemblance to other White middle-class ‘professionals’ that came to consult with them. JB has a Northen accent in contrast to the participants, and that might have helped the YP to see the JB as an outsider to other professionals they were familiar with. We knew it was important to spend time building trust to ensure that YP felt comfortable and confident in participating. We also assumed that they might find the subject of going to the doctors and dentists somewhat dry. We did not want the workshops to be experienced by YP as ‘extractive’, where YP felt that we were getting information with little return for them; we wanted YP to feel actively involved in and enjoy creating the content.

From JB’s previous experiences with seldom heard communities, the most honest and frank contributions often happen after a period of familiarisation and creativity. To encourage YP to feel confident in opening-in up, LH and JB spent at least 30 mins chatting informally with the participants before the start of each workshop. Participants had little prior knowledge of the researchers beyond the information the local facilitators conveyed – the purpose of the study and that it was led by people from a university – so these initial conversations were important in us getting to know one another. LH and JB demonstrated turn taking and listening in their own interactions. At every workshop, LH and JB shared lunch or dinner with participants during or after the workshop where they could get to know the researchers more informally, and these were relaxed and warm occasions.

To create a fun and engaging experience, JB structured the workshops to include fun, sometimes silly, microphone orientation tasks, true or false games, and monologue creation activities. These activities were unhurried and flexible enough to adapt to the needs of the YP, and they allowed participants to informally rehearse their thoughts and responses to the research questions. In one workshop, YP seemed particularly reluctant to get going at the start but once they were given the task of interviewing one another using handheld microphones, they quickly became engrossed in what they were doing. The informal conversations before the workshop combined with the creative activities helped to establish rapport and demonstrated that the facilitators were willing to listen. JB told the YP at the start of each workshop that whatever they shared was their gold; whatever they shared was valuable.

In three workshops, we think we managed to deliver a collaborative experience as YP were engaged, interactive, and relaxed throughout; however, in one workshop with only two participants (more were expected to attend), the adults initiated the dialogue throughout with YP less willing to expand naturally on their answers, perhaps because there were fewer YP than adults in the room. Unfortunately, as recruitment was challenging, postponing the workshop was not an option; instead, with hindsight, it may have been better for one of the facilitators to have stepped out.

There were some technical recording challenges arising from the settings we were in, for example, heavy rain on metal roofs, noisy kitchens, and larger than anticipated groups sharing microphones. Rather than continuously interrupting the YP to ask for a re-take (which would be standard practice when audio recording professional adults) JB prioritised the YP expressing themselves in the moment to ensure integrity of thought. Interruptions and re-takes would have undermined the trust in the workshop. Most of these environmental challenges have been removed or reduced in the podcast.

In relation to experiences that YP shared, JB, LH and EA anticipated that YP might feel uncertain or reticent about engaging with general practitioners, and like others, experience difficulties securing appointments. Having worked on a review of children and YP’s access, LH and EA expected that the YP may not feel they were being taken seriously or listened to well, though this was more surprising for JB. We could see that while YP understood what going to a local doctor or dentist meant, sometimes YP started to talk about seeing doctors at the hospital or an experience at A&E, indicating the term ‘primary care’ was abstract for some YP. LH, JB, and EA were struck by how conscious and empathic YP were of the pressure on medical services and professionals and their desire not to waste a professional’s time; however, this was at odds with their own experiences where they felt they could be perceived as a timewaster or a ‘tricky’ individual due to their more complicated needs. We were also struck by YP’s descriptions of the health reviews; it seemed strange to us to be regularly weighed and measured as teenagers, where physical development is a sensitive matter, without being told how that information was being used or offered a choice to not have it done. We noticed that older participants (18+) were more able to understand professionals as individual people, with their own quirks and expertise. Overall, we were moved by how clearly YP could articulate the challenges they faced and their own position in professional-patient interactions.

**The role of local facilitators**

Site A had a youth involvement group for young people currently in care and site B had two groups, one for young people in care and one for care leavers. The council facilitators had longstanding relationships with the young people in their groups and had several roles in facilitating the study. They selected and invited young people to participate, giving out our study advertisement and information sheets. Facilitators identified a suitable location for the groups and organised refreshments and vouchers. They sent out links to online consent forms and a consent explainer to young people and their carers (if participants were under 18) to complete in advance of the groups and ensure they were completed. Facilitators were available to provide practical and emotional support to participants during the groups.

**Data collection**

Before beginning the workshop, we checked each young person’s understanding of the study purpose, outlined the content of the session, and asked young people to complete a demographic form:

**Demographic form**

We are collecting a little bit of information about our participants so that we can describe who took part (without identifying you).

Please select the category that best applies to you or fill in where necessary. If you prefer not to answer any questions, leave them blank.

1. What is your gender? Please circle.
2. Female
3. Male
4. Trans
5. Non-binary
6. Other, please describe:_____________________
7. How old are you?
8. What is your ethnic background? Please circle.
   1. Asian or Asian British (Indian, Pakistani, Bangladeshi, Chinese, Any other Asian background)
   2. Black, Black British, Caribbean or African (Caribbean, African, Any other Black, Black British or Caribbean background)
   3. Mixed or Multiple ethnic groups (White and Black Caribbean, White and Black African, White and Asian, Any other Mixed or multiple ethnic background)
   4. White British (English, Welsh, Scottish, Northern Irish or British, Irish, Gypsy or Irish Traveller, Roma)
   5. White European or Other (Any other White background)
   6. Other ethnic group (Arab, Any other ethnic group)
   7. Prefer not to say
9. Who are you currently living with?
10. What age were you when you first were looked after?
11. How many placements have you had?

Ethnicity groups were based on UK Office for National Statistics categories, with the exception of adopting two separate categories for White British and White European or Other. These categories were separated based on consultation with a young person for a different research project on child health; we chose to collect the same demographics questions for both projects. In the manuscript, we have aggregated the categories to preserve participants’ anonymity.

Participants were given a ‘podcast preference statement’ which they could complete at any time during the session, which described the different ways that their voice could be incorporated into the podcast. All participants chose to use their own voice and a pseudonym.


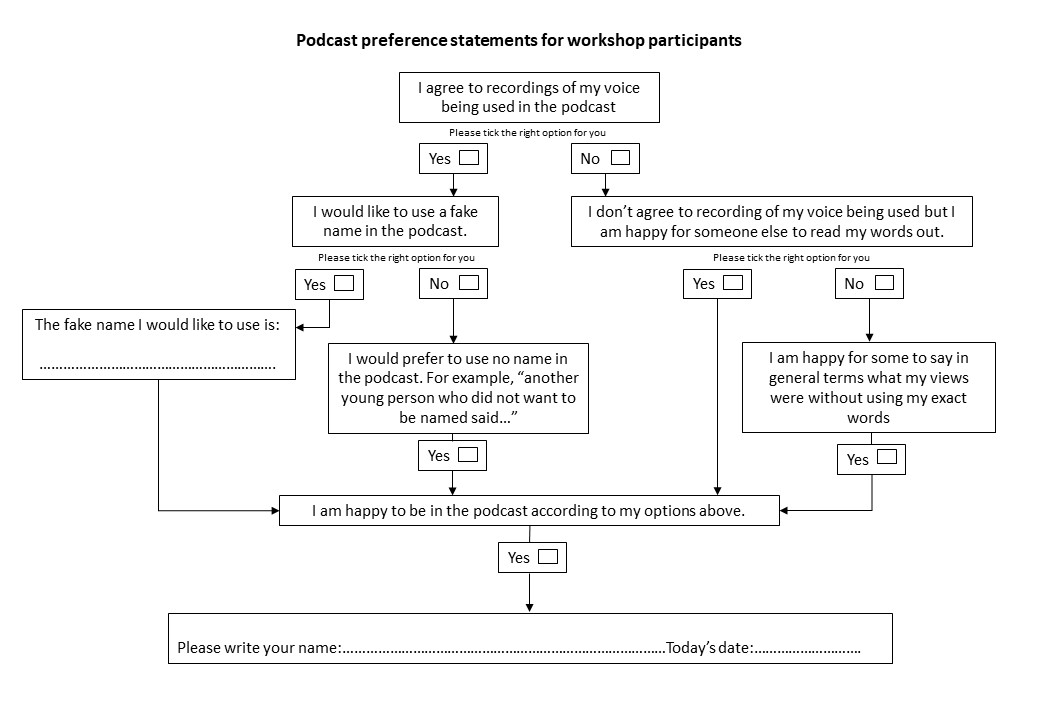


The workshop consisted of four core parts: an introduction, familiarisation with the microphone (where JB explained the purpose of each piece of recording equipment), a true/false game and discussion, and ideas from young people about improvements to healthcare. The option to script a monologue about a young person was used in one paired discussion where the creative facilitator thought participants would engage with the activity. In larger focus groups, participants were split into two smaller groups before the true/false game and given roaming microphones to rehearse their answers before rejoining for a full group discussion.

**Workshop plan**

**Part 1: Intros and notification of recording**

Participants to decide:

- Names – real name and fake name will be using.

Participants to answer:

- When was the last time you saw a doctor or nurse?
- When was the last time you saw a dentist?

(Prompt re if registered if it’s not clear)

**Part 2: Introduction to the microphone.**

Participants to complete script template and record it:

*My name is…*

*I’m here today to talk about…*

*A song I like is…*

*It makes me feel…*

*So we’re now going to listen to…*

*…hope you enjoy!*

**Part 3: True or False – horn of lies, bell of truth**

Participants to blow ‘horn of lies’ or ring ‘bell of truth’ and then talk about their answer.

- You have to be an adult to see a doctor on your own?
- It’s easy to get an appointment at the doctors or dentists.
- Mental health is equally important as physical health.
- It’s easy to get to the doctors.
- Doctors and nurses don’t judge people.
- I prefer to see the same doctor or dentist each time.
- I feel like I have enough time to talk about my problem when I’m at the doctors.
- I can talk to the doctor and nurse about anything (including things that are very private).
- People can talk to their doctor or nurse about relationship problems.

**Part 4: Monologue (optional)**

Participants to script monologue for a character.

*My name is…*

*No one knows but I’ve been feeling…*

*I’m worried because…*

*I’ve tried…*

*I just wish that…*

*What I’m going to do is…*

**Part 5: Change you want to see**

Ask participants:

- What could be improved for young people in being able to go to the doctors or dentists?
- Anything in particularly important for young people in care or coming out of care?

**Data analysis**

We used candidacy theory to provide a lens through which the data could be viewed to enhance the explanatory power of the analysis. We used to theory to inform the construction of higher-order themes, by examining our emerging themes and considering whether they were describing candidacy features. Initially, we had not identified the features ‘appearing at services’ and ‘permeability’ so LH re-examined the data to determine whether these features had coded under other features, or whether they were absent from the data. We found that we had placed data related to ‘appearing at services’ under ‘offers of, resistance to, services,’ as we had interpreted young people’s reluctance to speak to professionals’ as resistance to services rather than as difficulty in explaining needs. We did not find data relating to the permeability of services.

**Producing the podcast output**

The final workshops took place in April 2023. We aimed to produce a podcast output with content lasting around 15 mins from YP – we decided on this length to ensure we included depth of discussion but kept the audio accessible for busy professionals.

JB and LH met in May 2023 to carry out a day’s in-person side-by-side editing of the audio, akin to a rapid, descriptive analysis of emerging themes. The process of editing and producing the podcast audio is lengthy, and so the process of analysis to produce the podcast necessarily pre-dated the study’s analysis.

Prior to the editing, JB had repeatedly listened to the audio from the workshops, and LH had read the transcripts. JB and LH printed off the transcripts, cut out excerpts from each participant, breaking quotes down into meaningful sentences or paragraphs. We decided on the descriptive themes of: health reviews; history of the YP; mental health or relationships; instances of when health professionals had gone the “extra mile”; the experience of not being with family; seeing a GP with or without a carer; trust in professionals; feeling rushed at the doctors; and experiences of seeing a dentist. We organised the excerpts into an organic-sounding, narrative arc, removing quotes that were similar to reduce the audio length. JB edited the audio files accordingly to match the selected extracts. JB also added an introduction from each YP, “Hello, my name is X”, that we had recorded in each workshop.

The first draft of the audio was 27 mins, so we made further cuts to reduce the length. We excluded the data relating to health reviews and going to the dentists to keep the podcast more focused on general practice and relatable for the audience, for example, health reviews would require additional scaffolding from us as the hosts to inform listeners of the additional healthcare services provided for care-experienced YP.

When carrying out the side-by-side editing, LH and JB felt that the output would benefit from a response from healthcare professionals. LH and CP identified two healthcare professionals through their professional networks – a GP who is a child safeguarding lead and a community paediatrician – who were experienced in working with care-experienced or marginalised YP. The professionals agreed to listen to the rough cut of the YP’s audio and take part in a recorded online discussion about what YP had said. This discussion was recorded in September 2023. LH edited the transcript of the recording and JB then edited the audio. The audio from professionals was approx. 15 mins long.

On the same day as the recording with healthcare professionals, JB and LH recorded an intro to the podcast, outlining the study and contextualising the audio (that we had woven together recordings from different workshops in different environments). After the audio from YP and healthcare professionals was finalised, JB and LH met again in December 2023 to record the outro, summarising key points from the discussion, and adding credits.

The final podcast is called “Unheard voices: young people in care's experiences of speaking to doctors” and can be accessed here: <https://mediacentral.ucl.ac.uk/Play/110455>.
